# Extraction of Hydroxyapatite Nanostructures from Marine Wastes for the Fabrication of Biopolymer-Based Porous Scaffolds

**DOI:** 10.3390/md18010026

**Published:** 2019-12-27

**Authors:** Hengameh Gheysari, Fatemeh Mohandes, Mozhdeh Mazaheri, Banafsheh Dolatyar, Masoud Askari, Abdolreza Simchi

**Affiliations:** 1 Department of Materials Science and Engineering, Sharif University of Technology, International Campus, P.O. Box 79417-76655, Kish Island, Iran; hengamehgheysari@gmail.com; 2 Department of Materials Science and Engineering, Sharif University of Technology, P.O. Box 11155-9161, Azadi Avenue, Tehran 14588, Iran; mohandes1120@gmail.com (F.M.); mazaheri.mozhdeh@gmail.com (M.M.); askari@sharif.edu (M.A.); 3 Department of Cell and Developmental Biology, School of Biological Sciences, College of Science, University of Tehran, P.O. Box 14155-6619, Tehran, Iran; banafsheh.dolatyar@gmail.com; 4 Institute for Nanoscience and Nanotechnology, Sharif University of Technology, P.O. Box 11365-9466, Azadi Avenue, Tehran 14588, Iran

**Keywords:** marine waste, biopolymer, tissue engineering, cytotoxicity

## Abstract

Three-dimensional porous nanocomposites consisting of gelatin-carboxymethylcellulose (CMC) cross-linked by carboxylic acids biopolymers and monophasic hydroxyapatite (HA) nanostructures were fabricated by lyophilization, for soft-bone-tissue engineering. The bioactive ceramic nanostructures were prepared by a novel wet-chemical and low-temperature procedure from marine wastes containing calcium carbonates. The effect of surface-active molecules, including sodium dodecyl sulfate (SDS) and hexadecyltrimethylammonium bromide (CTAB), on the morphology of HA nanostructures is shown. It is demonstrated that highly bioactive and monophasic HA nanorods with an aspect ratio > 10 can be synthesized in the presence of SDS. In vitro studies on the bioactive biopolymer composite scaffolds with varying pore sizes, from 100 to 300 μm, determine the capacity of the developed procedure to convert marine wastes to profitable composites for tissue engineering.

## 1. Introduction

Hydroxyapatite (HA), which belongs to the calcium phosphate family, has attracted much attention to disclose a new class of hybrid materials integrating mechanical properties and biological activity [1,2,3]. HA is biocompatible with surface-active living tissues and possesses chemical and physical similarities to the inorganic phase of human bone and teeth [4,5,6]. Various forms of HA have been employed applied to modify the surface metallic implants [7], fill and repair bone defects in tissue engendering [8], and conduct protein purification [9].

So far, many attempts have been made to use marine-derived materials to prepare biomaterials [10,11,12,13]. For example, HA nanostructures have been synthesized from natural biowastes, such as skeletons of marine organisms like corals [14], mussel [15], oyster [16], and crab shells [17] via hydrothermal, microwave, and thermal treatment methods. Although the commercial calcium sources are widely used to prepare HA nanostructures [18,19], comprehensive availability, low cost, and biological-natural origin of biowastes are highly attractive properties for the fabrication of HA [20,21]. On the other hand, HA synthesized from natural sources has shown better tissue response and easily bonds with human bone [22]. Thus, a novel surfactant-assisted precipitation method using oyster shells as a calcium source is presented, here, to prepare HA nanoparticles and nanorods. The prepared nanostructures were then used as bioactive phase, to develop biopolymer-based tissue-engineering scaffolds.

For tissue-engineering applications, porous scaffolds with 3D structure should be used to mimic the role of extracellular matrix (ECM) [23]. To design and preparation of three-dimensional (3D) scaffolds, hydrogels based on biopolymers such as gelatin (Gel) and chitosan (CS) [24,25,26], collagen [5,27,28,29,30], sodium alginate [31], bacterial cellulose [32], as well as carboxymethyl cellulose (CMC) [33], have been wieldy used due to their biocompatibility and biodegradability. Among these biopolymers, gelatin retains specific collagen information signals; an example of this is arginyl-glycyl aspartic acid (RGD) tripeptide, which can promote cell-adhesive activity to some extent [34]. In addition, CMC as a natural polysaccharide may react with some molecules due to its electric charge [35,36]. These polymers also exhibit a high swelling ratio when immersed in PBS [37]. Therefore, they are promising candidates to fabricate 3D porous scaffolds.

In vascularized tissues, the amount and size of pores affect the diffusion of oxygen and nutrients, waste removal, and angiogenesis [38,39]. Interconnected pore networks provide an effective platform for the release of proteins, drugs, and growth factors [40,41,42]. The mechanical properties of porous structures are also influenced because the mechanical integrity and structural stability of the scaffolds are determined by the pore structure [43]. Among different techniques developed for the fabrication of porous scaffolds, such as salt leaching, gas foaming, electrospinning, and freeze-drying [39], we employed a combination of salt-leaching and lyophilization technique. While the lyophilization technique can form highly interconnected and uniform pore structure, salt leaching creates pores with required sizes for practical applications (e.g., 100–350 µm for bone regeneration, 40–100 µm for osteoid ingrowth, and 5–10 µm for vascularization) [39]. Highly porous nanocomposites of HA nanorods, gelatin (Gel), and carboxymethyl cellulose (CMC) hydrogels cross-linked with citric acid (CA) were prepared. The potential application of the materials derived from marine biowastes for bone-tissue engineering is demonstrated via in vitro studies.

## 2. Results

### 2.1. Characterization of HA Nanostructures

**Figure 1:**
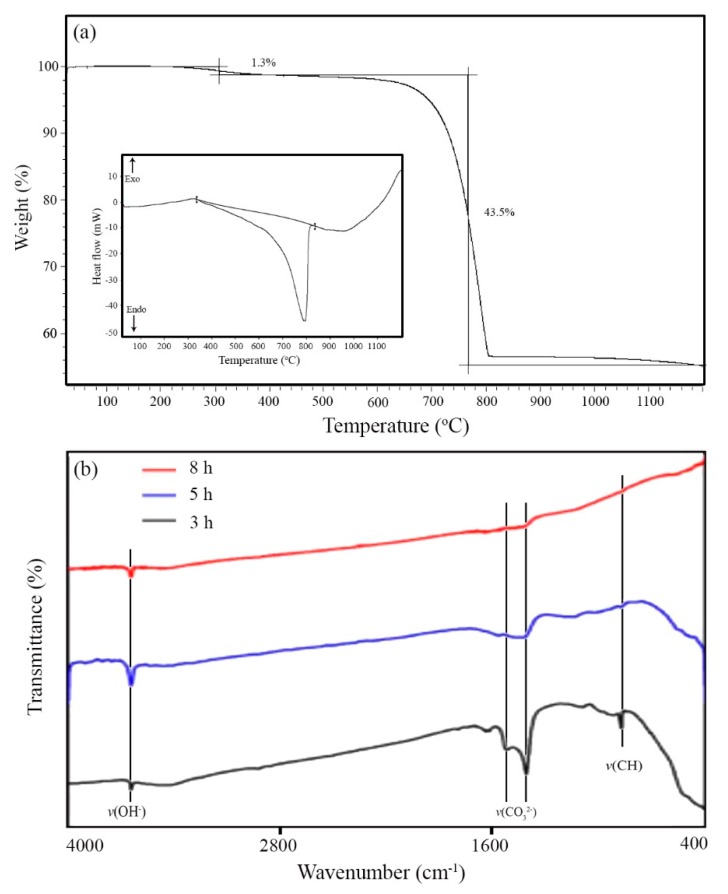
(**a**) TGA and DSC (inset) curves of oyster shell powders heated in air. (**b**) FTIR spectrum of the calcined shell at 800 °C for different times.

Seashells typically consist of 95–99% CaCO_3_ and 1–5% organic materials (proteins and glycoproteins) [44]. In order to remove the organic materials and obtain calcium oxide, the oyster shell powder was calcined at 800 °C. Thermal gravimetric (TGA) and differential scanning calorimetry (DSC) curves are presented in Figure 1a. The results revealed that the organic phase was removed at around 400 °C, and CaCO_3_ was converted to CaO in the range of 760–800 °C. TGA curve indicated two-step weight loss with an amount of 1.3% and 43.5%, which correspond to organic phase removal and calcium carbonate decomposition, respectively.

For complete removal of the organic phase from the oyster shell, calcination of the powder was performed at 800 °C for different times (3, 5, and 8 h). The FTIR spectrum of the products is illustrated in Figure 1b. The stretching vibration peaks of *v*(CO_3_^2−^) and *v*(CH_2_) appeared at 1545 at 1415 cm^−1^, respectively. The adsorption bands in the range of 3100–3500 cm^−1^ (stretching vibration of OH) and 1630–1640 cm^−1^ (bending vibration of OH) are attributed to the surface-adsorbed water molecules [19,45]. The results indicated that calcination for 8 h removed all organic materials.

**Figure 2:**
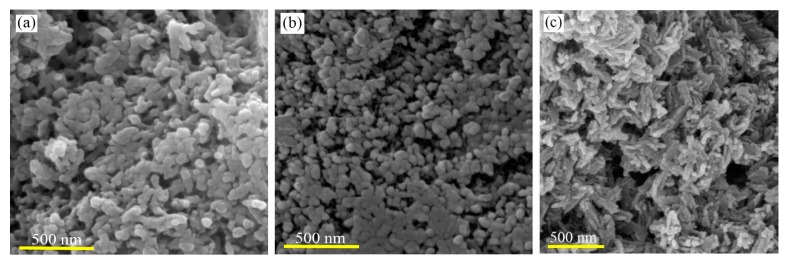
FESEM images of HA nanostructures synthesized (**a**) without adding surfactant and with introducing (**b**) CTAB and (**c**) SDS.

The morphology and particle size of biowaste-derived HA were studied by SEM (Figure 2). Without surfactant, agglomerated particles with irregular shapes and wide sizes were obtained (Figure 2a). To avoid particle agglomeration, CTAB and SDS were used. In the presence of CTAB, particles with sizes in the range of 50–80 nm were synthesized (Figure 2b). When SDS was used, rod-like morphologies of HA were obtained (Figure 2c). The nanorods had dimensions of 15–25 nm (diameter) and 100–150 nm (length).

**Figure 3:**
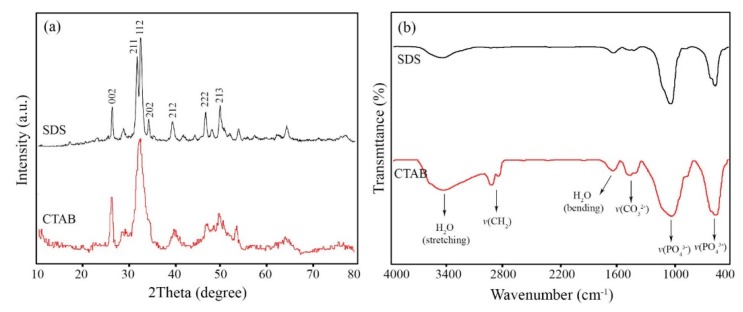
(**a**) XRD patterns and (**b**) FTIR spectrum of HA nanostructures synthesized in the presence of surfactants.

XRD patterns of the HA nanostructures synthesized in the presence of SDS and CTAB are shown in Figure 3a. All the diffraction peaks can be indexed to hexagonal phase hydroxyapatite (JCDPS No. 72-1243). The diffraction peaks at 2θ values (degree) of 25.851, 31.781, 32.221, 32.871, 34.041, 39.811, 46.691, and 49.521 are attributed to (002), (211), (112), (300), (202), (130), (222), and (213) crystal planes, respectively. No diffraction peaks related to other calcium phosphate phases like Ca_3_(PO_4_)_6_ could be detected. The XRD pattern of HA nanoparticles formed by CTAB shows a composite peak consisted of (211) and (112) planes at 2θ = 32°. This finding reveals the lower crystallinity of the nanoparticles than the nanorods. Based on the method explained in [46], the crystallinity degree of the HA nanostructures prepared by SDS and CTAB was about 27% and 24%, respectively.

Figure 3b displays FTIR spectrum of the HA nanostructures. The adsorption band located at 471 cm^−1^ corresponds to the symmetric stretching vibrations (*v*_2_) of PO_4_^3−^, while the bands at around 567–603 cm^−1^ are attributed to its bending vibrations (*v*_4_). The bands centered at around 900–1200 cm^−1^ are derived from the symmetric and asymmetric stretching vibrations (*v*_1_ and *v*_3_) of PO_4_^3−^ [18]. The broad band at 3100–3400 cm^−1^ (stretching vibration) and weak band at 1630–1635 cm^−1^ (bending vibration) are attributed to the surface adsorbed water molecules. The presence of the *v*(CO_3_^2−^) vibrations at around 1415–1458 cm^−1^ were also noticed. The origin of the carbonate ions might be the carbon dioxide in the atmosphere, which is absorbed during the dissolving and stirring processes [19]. Besides, the stretching vibrations of CH_2_ bands at 2958 and 2850 cm^−1^ indicate the presence of CTAB molecules on the surface of the nanoparticles [47]. In contrast, SDS could easily be removed from the surface through the washing process.

### 2.2. Microstructure of HA-Biopolymer Scaffolds

**Figure 4:**
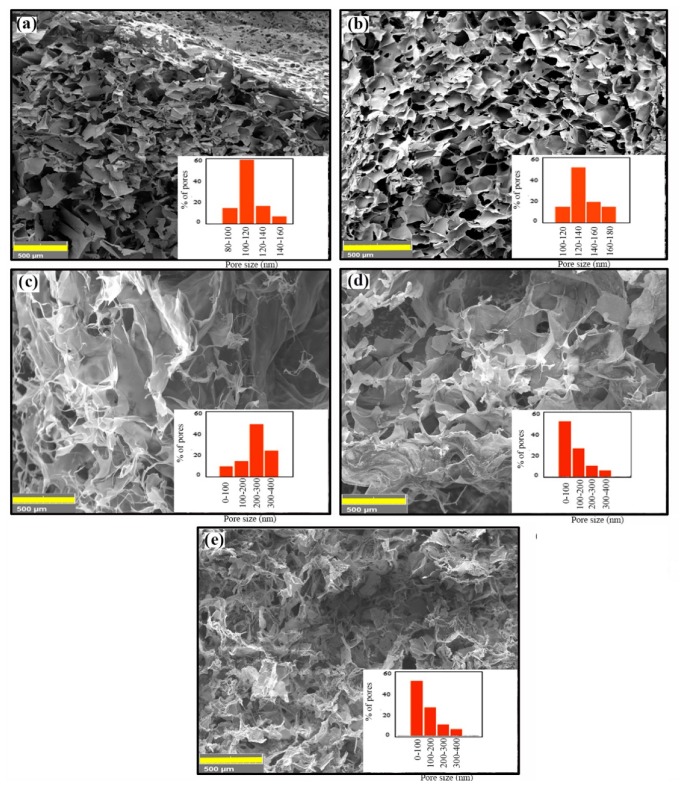
FESEM images of HA/Gel/CMC scaffolds synthesized by (**a**) 0.025 g CA, (**b**) 0.25 g CA, (**c**) NaCl (420–500 µm), (**d**) NaCl (180–250 µm), and (**e**) NaHCO3. The pore-size distribution was shown in the inset of each image.

Cross-sectional SEM images of HA/Gel/CMC scaffolds cross-linked by 0.025 and 0.25 g of CA are shown in Figure 4a,b respectively. The scaffolds have a spongy-like microstructure with relatively uniform pore structure. The size of pores depends on the concentration of CA and varies in the range of 80−150 μm (for 0.025 g of CA) and 120−180 μm (for 0.25 g CA). Since larger pores are required for bone tissue engineering (100 to 350 μm [39]), salt-porogen strategy was used. The effect of porogen size on the pore structure of the scaffolds cross-linked by 0.25 g of CA was studied by SEM. Figure 4c shows the microstructure of the scaffolds synthesized in the presence of NaCl (180−250 μm). Larger pores were formed while the pore walls became thinner. When coarser NaCl particles (420−500 μm) were used, the size and interconnectivity of the pores were improved (Figure 4d). NaHCO_3_ particles (200−250 μm) resulted in the formation of micro-sized pores, as well as very fine pores on the pore walls (Figure 4e). The amount of micro-size pores was lower than that of NaCl-treated specimen. The formation of fine pores can be ascribed to the decomposition of calcium carbonate and gas release very similar to the mechanism of pore formation in the gas-foaming technique [48]. The effect of porogen on the size distribution is shown in Figure 4f.

### 2.3. Crosslinking of HA-Biopolymer Scaffolds

**Figure 5:**
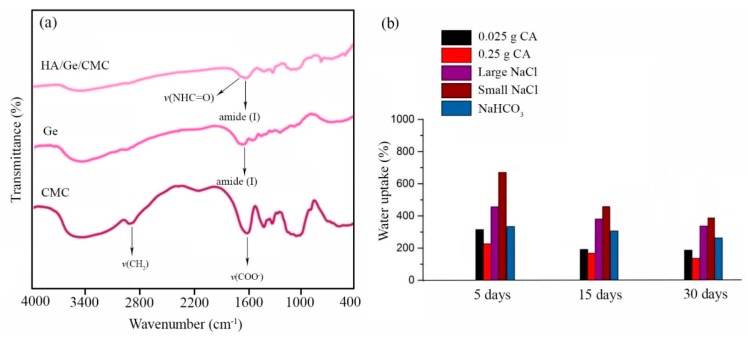
(**a**) FTIR spectrum of gelatin, CMC, and HA/Gel/CMC scaffolds. (**b**) Water uptake of HA/Gel/CMC scaffolds as a function of soaking time.

FTIR spectroscopy was used to confirm the crosslinking of Gel and CMC chains by CA. The spectra of the examined materials are shown in Figure 5a. The IR spectrum of CMC shows bands at 3404 and 3200 cm^−1^ due to the O–H stretching vibrations [37]. The adsorption bands at 2918 and 2907 cm^−1^ are related to the aliphatic C–H stretching vibrations, but those appearing at 1618 and 1420 cm^−1^ are attributed to the asymmetric and symmetric stretching of the carboxylate group, respectively. The bands found at 1108 and 1060 cm^−1^ represent C–O–C stretching vibrations [33]. In the FTIR spectrum of Gel, a characteristic band due to N–H stretching is observed at 3413 cm^−1^ [49]. The N–H bending vibration is indicated by a band observed at 1539 cm^−1^. Aliphatic C–H stretching and bending vibrations are visible at 2850–2940 and 1400–1450 cm^−1^, respectively. The band appearing at 1641 cm^−1^ indicates amide (I) band, while bands at 1335 and 1238 cm^−1^ indicate the C–N stretching vibrations [50]. In the FTIR spectrum of the cross-liked HA/Gel/CMC scaffolds, characteristic peaks of Gel and CMC are observed. Additionally, the stretching vibration of the amid group (–NHC=O–) at around 1700 cm^−1^ indicates cross-linking between the polymer chains.

### 2.4. Water Uptake of the Scaffolds

Figure 5b displays the water uptake of the scaffolds in PBS (pH = 7.4) at 37 °C, up to 30 days. It was found that the water uptake of the scaffolds cross-linked by different amounts of CA and without the addition of porogen particles was not very different (about 200–300%). Processing with porogen particles increased the water uptake capacity of the scaffolds. Smaller NaCl particles had more effect on the water uptake than the larger ones. The highest water uptake (>600%) was attained for the porous scaffolds prepared by the finer fraction of NaCl particles.

### 2.5. Mechanical Strength of 3D Scaffolds

**Figure 6:**
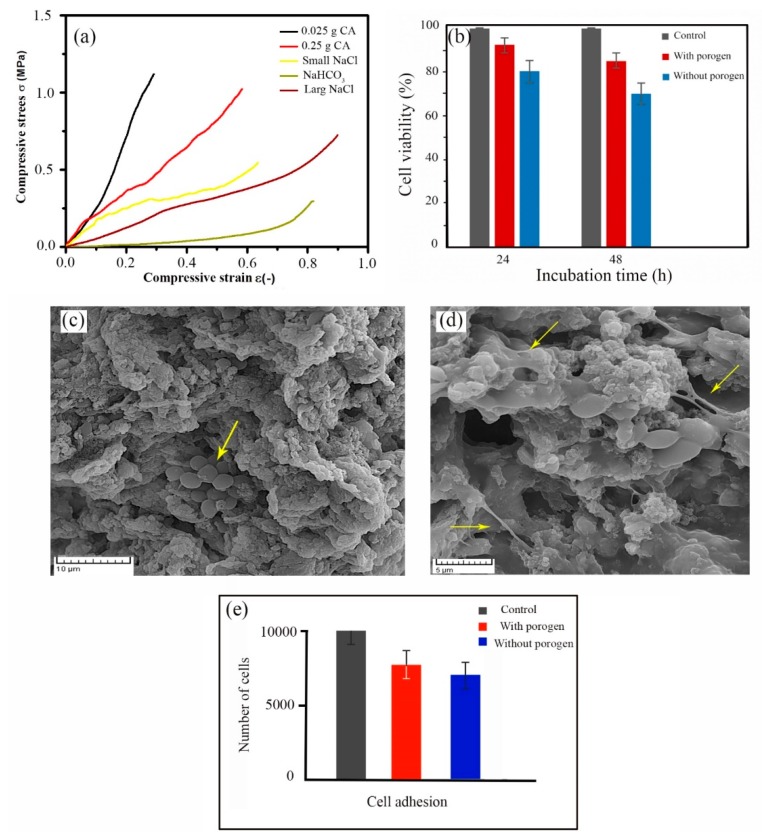
(**a**) Compressive stress–strain curves of HA/Gel/CMC scaffolds cross-linked by different concentrations of CA and porogens. (**b**) Cell viability of HA/Ge/CMC scaffolds synthesized with NaCl (180–250 µm) and without porogens. FE-SEM images of the cell-cultured scaffolds prepared (**c**) without porogen and (**d**) with NaCl particles (180–250 µm). (**e**) The number of adhered cells on scaffolds after 4 h incubation.

Typical stress–strain curves of the HA/Gel/CMC scaffolds cross-linked by CA at different concentrations with and without salts are shown in Figure 6a. Each of the experimental points reported in this analysis is an average value of three measurements. The measured compressive modulus is reported in Table 1. The modulus varied in the range of 1.5 to 2.8 MPa, depending on the processing condition, which determines the pore structure. The addition of porogen particles increased the size and volume of pores, thereby reducing the mechanical durability in agreement with previous studies [24,43].

### 2.6. In Vitro Biocompatibility Studies

The cytotoxicity of the scaffolds was evaluated by MTT assay. Figure 6b shows the high viability of MG63 osteoblast-like cells incubated on the scaffolds synthesized with the help of NaCl particles (180–250 µm) as compared with the control. Cell viability of the scaffolds fabricated without porogen particles is lower than the scaffolds fabricated with NaCl at the same conditions. This observation may be related to the pore structure of the scaffolds, which play a key role to cell attachment and growth [51]. Figure 6c,d shows SEM images of the cells grown on the scaffolds synthesized without and with NaCl porogen (180–250 µm). The MG-63 cells attached to the scaffolds was noticed. Quantitative analysis of cell adhesion (Figure 6e) determined that about 85% and 72.5% of the cells seeded were adhered to the surface of the scaffolds synthesized with and without porogen, respectively.

## 3. Discussion

**Figure 7:**
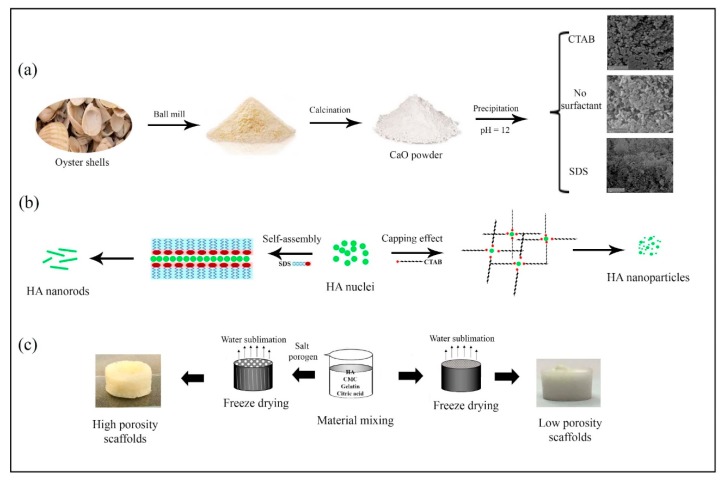
Schematic presentation of (**a**) HA synthesis from oyster shells, (**b**) effect of surfactants on the HA morphology and particle size, and (**c**) effect of freeze-drying and salt-porogen on the scaffolds’ porosity.

The aim of this work relies on the extraction and preparation of HA nanostructures from marine biowastes to develop HA/Gel/CMC composites as bone scaffolds. The developed procedure used for the HA synthesis from biowastes is schematically summarized in Figure 7a. First, the oyster shell powder containing CaCO_3_ was heated at 800 °C for 8 h, to remove organic phases and to obtain CaO powder. Second, HA nuclei were gradually formed and grown through a wet-chemical precipitation method by dropwise adding NaOH solution into the mixture containing H_3_PO_4_ and the extracted CaO. As seen in Figure 2a, agglomerated and micro-size particles with irregular shapes were produced through the precipitation method, without using any surfactant. In order to control the growth process, the effect of ionic surfactants on the size and morphology of the HA nuclei was investigated. When CTAB as cationic and SDS as anionic surfactants were used, sphere-like and rod-like HA nanostructures were obtained, respectively (Figure 2b,c).

At surfactant concentrations above the critical micellar concentration (CMC), CTAB and SDS molecules form micelles [52]. We used an SDS concentration (8.3 mM) close to its CMC (~8 mM [53]), hence, lamellar micelles with negative surface charges are formed [53]. Interactions of negative surface charges of SDS with Ca^2+^ ions located at the columns and corners of the HA hexagonal unit cells [54] lead to the fusion and self-assembly of the nuclei in an oriented manner, forming HA nanorods. Utilizing CTAB with a concentration of 6.5 mM, which is far away from CMC (~1 mM [55]), results in the formation of irregular-shaped HA nanoparticles. This finding reveals that, at high CTAB concentrations, micelles do not act as a template for the HA growth. However, the steric hindrance effect of the surfactant limits the particle growth, so that finer HA particles are formed. Similar results have been observed by Wu et al. [56] about the synthesis of Cu nanoparticles in the presence of very high concentration of CTAB as capping agent. The proposed formation mechanism of the HA nanostructures was schematically illustrated in Figure 7b.

The fabricated HA nanorods with high bioactivity and structural similarity to the inorganic phase of bone and teeth [57] were used to prepare highly porous HA/Gel/CMC scaffolds by combined salt-porogen and freeze-drying techniques. The fabrication process of the porous HA/Gel/CMC scaffolds with and without salt-porogen is schematically seen in Figure 7c. The aim of utilizing porogen particles was tuning the pore sizes to the range suitable for bone regeneration [39]. It was shown that coarser porogen particles create larger pores, with better pore interconnectivity. The interconnectivity of the scaffolds’ pores affects not only the transport of oxygen and nutrients into and waste out of the cells, but also other scaffold properties like water-absorption capacity, facilitating the protein absorption that are required for bone regeneration [58]. Based on the SEM images presented in Figure 4e, the use of NaHCO_3_ particles (200–250 μm) resulted in the formation of micro-sized pores (100–300 μm), as well as very fine pores on the pore walls. The formation of very fine pores can be attributed to the decomposition of calcium carbonate and gas release, corresponding to the mechanism of pore formation in the gas-foaming technique [48].

Overall, this study indicated that varying the porosity and pore sizes effectively altered the mechanical properties of the HA/Gel/CMC composites. Using coarser porogen particles increases the porosity level, whereas it negatively affects the mechanical strengths. As seen in Table 1, compressive modulus of the scaffolds modified by the porogens is lower than that of the scaffold synthesized without the porogens. Generally, the porogens increase void space or porosity, thinner walls, and disconnected solid network, which significantly weakens compressive strength of the scaffolds [59,60]. As a result, compressive strength and porosity were inversely proportional. Because of the poor mechanical properties of the highly porous HA/Gel/CMC scaffolds, these materials are introduced as bone grafts to repair non-load-bearing bones.

In vitro cytotoxicity assay of the scaffolds illustrated in Figure 6b shows that proliferation of the cells on the scaffolds is lower than that of the control test after 24 and 48 h of cell incubation. When the cells at high densities (1 × 10^4^ cells per scaffold) are distributed on the scaffolds, the pores are rapidly filled by the cells, decreasing the cell proliferation due to cell contact growth inhibition [51]. Figure 6b also indicates the greater number of cells (~93%) are observed during the proliferation period on the scaffolds prepared by the NaCl porogen. The high porosity and interconnectivity of the pore network allow cells to penetrate into the scaffolds while facilitating oxygen and nutrients transportation [61,62]. In vitro cell studies (Figure 6) also determined that multiple filopodia were extended from the cells to the scaffolds fabricated by NaCl porogen (Figure 6d). When porogen particles were not used, more spherical cells were observed (Figure 6c). The numbers of the adhered cells on the scaffolds was also affected by the porogen particles (Figure 6e). Therefore, it is suggestible that the pore structure, including their size and interconnectivity, plays a key role in cell attachment.

## 4. Materials and Methods

### 4.1. Materials

Oyster shells were collected from Persian Gulf beach, Iran. Sodium dodecyl sulfate (SDS, 85%, Merck, Kenilworth, NJ, USA), hexadecyltrimethylammonium bromide (CTAB, Sigma-Aldrich, St. Louis, MI, USA), gelatin (Gel, for microbiology, Type A, Bloom 300, Merck), citric acid (99.5–100.5%, Merck), sodium carboxymethyl cellulose (CMC, MW = 90,000 Da, Merck), phosphoric acid (85%, Merck), sodium hydroxide (97%, Merck), sodium chloride (99.5%, Merck), potassium chloride (99.55%, Merck), disodium hydrogen phosphate (99.0%, Merck), dihydrogen potassium phosphate (99.5–100.5%, Merck), sodium hydrogen carbonate (99%, Merck), and biocompatible grade NaCl with two different size ranges (180–250 and 420–500 μm) were used without further purification.

### 4.2. Preparation of HA Nanostructures

The developed procedure for extracting HA from biowastes like teeth was employed with moderate modification [63]. The as-collected oyster shells were cleaned with distillated water and ethanol, using a brush and sonication (15 min). After drying, the shells were ground in a disk mill, and the finer fraction was collected by a 65-mesh sieve. The powder was then calcined at 800 °C for 8 h in air, to completely remove the organic materials and impurities. HA nanostructures were synthesized by reaction between the calcium carbonate extracted from the oyster shell powders and phosphoric acid (H_3_PO_4_), in the presence of SDS and CTAB as surfactant. In a typical procedure, 0.1 g of CaCO_3_ was dispersed into 50 mL of DI water by sonication for 15 min. Then, an appropriate amount of H_3_PO_4_ was added dropwise into the suspension, to obtain a clear solution with continuous stirring, using a magnetic stirrer at room temperature. Afterward, 0.12 g of the surfactant was added into the solution, with stirring for 60 min. In order to form HA nuclei, NaOH solution (1.0 M) was added dropwise into the solution to maintain the pH at 12. The pH value of the mixture was kept at 12, and the mixture was refluxed at 90 °C for 4 h. The obtained precipitate was collected by centrifuge, washed several times with DI water and ethanol, to remove possible residuals of acid and the surfactant, and finally dried in an oven at 80 °C for 6 h.

### 4.3. Preparation of HA-Biopolymer Scaffolds

Highly porous nanocomposite scaffolds of HA-Gel/CMC were prepared by lyophilization. In a typical procedure, HA nanorods were dispersed in an aqueous solution of Gel (5 wt.%) and CMC (3 wt.%) by sonication for 15 min. The crosslinker was then added into the mixture, and the suspension was stirred for 24 h. To remove the excess amount of the cross-linker, dialysis (12 KDa bag dialysis) against DI water for 24 h was performed. The pH value of the mixture was 7. NaCl particles with two size distribution range (180–250 and 420–500 μm) and NaHCO_3_ (420–500 μm) were used as porogen. The mass ratio of Gel to porogen was 1:1. Following a stirring process, the homogenized mixtures containing of Gel, CMC, HA, and NaCl were poured into 12-well cell culture plates, and then the cross-linker was added. The mixture was left for 24 at room temperature and then lyophilized by an Alpha 2-4, Martin Christ freeze-dryer (Osterode, Germany) at −50 °C and 1.0 mbar. To remove the porogen, the lyophilized specimens were immersed in DI water for 12 h. The solvent was refreshed every 3 h. Finally, porous scaffolds were obtained by lyophilization.

### 4.4. Materials Characterizations

Fourier transform infrared (FT-IR) spectrum of the products was recorded on a Magna-IR, spectrometer 550 Nicolet in KBr pellets in the range of 400–4000 cm^−1^. Micrographs of HA powders were taken by using a field-emission scanning electron microscope (FE-SEM, TESCAN, Brno, Czech Republic). Powder X-ray diffraction (XRD) patterns were collected from a diffractometer of Philips Company (Amsterdam, The Netherlands) with X’Pert Pro monochromatized Cu *K*α radiation (λ = 1.54 Å, operated on 35 mA and 40 kV current).

For microstructural studies, the porous scaffolds were frozen at −50 °C and cross-sectionally cut to small pieces by a sharp surgical blade. After gold sputtering, the cross-sections were observed by SEM. Pore size distribution of the porous scaffolds were supplied by Digimizer software. Thermogravimetric analysis (TGA) and derivative thermogravimetry (DTG) were carried out with Pyris Diamond Perkin Elmer, under air atmosphere, at a heating rate of 10 °C/min, from room temperature to 1200 °C.

### 4.5. Mechanical Properties

To assess the mechanical properties of the HA/Gel/CMC scaffolds, a uniaxial compression test was performed at room temperature by a universal testing machine (SHIMADZU, AGS-J, Japan) at a cross-head speed of 1 mm/min. Cylindrical-shaped specimens with a diameter of 25 mm and height of 15 mm were examined. The elastic modulus was calculated as the slope of the initial linear portion of the stress–strain curve. For each specimen, the compression tests were repeated three times, and the average of the results was reported with standard deviation.

### 4.6. Water Uptake

To determine the amount of water uptake, the weight of the specimens before (*W*_i_) and after immersion (*W*_w_) in PBS (pH = 7.4; 37 °C) was measured. After selected time intervals (5, 15, and 30 days), the specimens were taken out from the medium and the excess water was removed. The amount of water uptake was calculated by the following equation [43]:

*W*_L_ = (*W*_w_ − *W*_i_)/*W*_i_ × 100% (1)

### 4.7. Porosity

The amount of total porosity of the scaffolds was determined by the volumetric method [64]. Cylindrical-shaped scaffolds were prepared and weighted by an accurate balance (CP324S model, Sartorius, Germany). The volume was determined by measuring the dimensions by a micrometer. The total porosity was then calculated from the ratio of the scaffold density per the pore-free density.

### 4.8. Cell Viability, Attachment, and Adhesion

In vitro cell viability of the HA/Gel/CMC scaffolds was measured using MTT (3-(4,5-dimethylthiazol-2-yl)-2,5-diphenyltetrazolium bromide) assay, which is based on the mitochondria reduction of MTT to form an insoluble dark blue formazan product. This reduction takes place only when mitochondrial reductase enzymes are active. To obtain the extracts of the scaffolds, the samples were incubated at 37 °C in 1 mL of RPMI 1640 culture medium (Sigma) supplemented with 10% (*w*/*w*) fetal bovine serum (FBS), for 24 and 48 h. The culture medium (RPMI and FBS) under similar condition was used as the control. The cells were seeded into 96-well plates at a density of 1 × 10^4^ cells per scaffold. The culture medium was replaced with the extract of the scaffolds. After 24 h, the extract was eliminated, and 100 mL of the MTT solution (0.5 mg/mL) was added to each well, and the cells were incubated at 37 °C for an additional 4 h. The solution was then removed, and 100 µL of isopropanol was subsequently added to dissolve the MTT crystals. The absorbance of the solutions was quantified by spectrophotometer at 570 nm, with a microplate reader (Power wave XS2, BioTek, Winooski, VT, USA).

Morphology of the attached cells on the scaffolds was observed by FE-SEM. For this purpose, the scaffolds were placed in Petri dish and incubated with MG-63 cells in DMEM for 48 h at 37 °C. After incubating, the scaffolds were washed several times with PBS and fixed with glutaraldehyde solution (2.5%) at 4 °C for 4 h. Finally, the specimens were air-dried and then gold-coated for FE-SEM studies. The density of the cells seeded into 96-well plates were about 1.0 × 10^4^ cell/scaffold. The numbers of the adhered cells to the scaffolds were estimated after 4 h of incubation by hemocytometer.

### 4.9. Statistical Analysis and Codes of Ethics

Statistical Package for the Social Sciences (SPSS software, Version 16, IBM, Armonk, NY, USA) was used for one-way ANOVA (*p* < 0.05, *n* = 5), to evaluate the statistically significant difference between measurements.

## 5. Conclusions

Monophasic HA nanostructures were prepared by oyster shell powders as precursor via a facile precipitation method. The particle sizes and morphology of HA were controlled in the presence of SDS and CTAB surfactants. It was shown that HA nanorods could be synthesized from milled and calcined powder (800 °C for 8 h) in the presence of SDS. The nanorods had a dimeter of 15–25 nm and length of 100–150 nm. Highly porous scaffolds of Gel/CMC hydrogels and HA nanorods were then prepared by lyophilization and porogen technique. It was found that NaCl with particle sizes of about 180–250 µm led to the fabrication of uniform pours scaffolds with pore sizes in the range of 200–300 µm. Although porosity and pore size of the scaffolds showed an upward trend by increasing the size of the porogens, the compressive modulus was decreased. Aside from this, the water uptake properties of the scaffolds depended on the type and size of porogen, and the highest amount (>600%) was attained for the scaffolds prepared in the presence of NaCl with particle sizes of 180–250 µm. In vitro studies indicated that the biodegradable HA/Gel/CMC nanocomposites were not toxic (cell viability > 80% after 48 h of incubation). An effective improvement in cell attachment and adhesion was attained by employing porogen, causing the pores with sizes in the range of 200 to 300 µm. Therefore, the porous nanocomposite scaffolds are suitable substitutes for repairing non-load bones.

## Figures and Tables

**Table 1:** Microstructural features and mechanical strength of HA/Gel/CMC scaffolds.

Sample	CA Concentration (g)	Porogen	Pore Size (µm)	Porosity (%)	Compressive Modulus (MPa)
S1	0.025	-	80–200	81.5 ± 5.5	2.6 ± 0.5
S2	0.25	-	100–150	80.2 ± 3.7	2.8 ± 0.3
S3	0.25	NaCl (180–250 µm)	200–300	92.5 ± 3.5	1.5 ± 0.5
S4	0.25	NaCl (420–500 µm)	100–200	93.5 ± 4.2	1.6 ± 0.7
S5	0.25	NaHCO_3_ (200–250 µm)	100–300	92.3 ± 3.7	1.2 ± 0.4

## References

[1] Dorozhkin S.V. (2011). Calcium orthophosphates: Occurrence, properties, biomineralization, pathological calcification and biomimetic applications. Biomatter.

[2] Dorozhkin S.V. (2015). Calcium orthophosphate deposits: Preparation, properties and biomedical applications. Mater. Sci. Eng. C Mater. Biol. Appl..

[3] Dorozhkin S. (2017). A history of calcium orthophosphates (CaPO4) and their biomedical applications. Morphologie.

[4] Ehrlich H., Douglas T., Scharnweber D., Hanke T., Born R., Bierbaum S., Worch H. (2005). Hydroxyapatite crystal growth on modified collagen I-templates in a model dual membrane diffusion system. Z. Anorg. Allg. Chem..

[5] Ehrlich H. (2010). Chitin and collagen as universal and alternative templates in biomineralization. Int. Geol. Rev..

[6] Ehrlich H. (2010). Chapter 3, Biocomposites and mineralized tissues, In Biological Materials of Marine Origin.

[7] Łukaszewska-Kuska M., Krawczyk P., Martyla A., Hędzelek W., Dorocka-Bobkowska B. (2018). Hydroxyapatite coating on titanium endosseous implants for improved osseointegration: Physical and chemical considerations. Adv. Clin. Exp. Med..

[8] Moeini S., Mohammadi M.R., Simchi A. (2017). In-situ solvothermal processing of polycaprolactone/hydroxyapatite nanocomposites with enhanced mechanical and biological performance for bone tissue engineering. Bioact. Mater..

[9] Jeon M., Jung S., Park S. (2018). Facile covalent bio-conjugation of hydroxyapatite. New J. Chem..

[10] Diaz-Rodriguez P., López-Álvarez M., Serra J., González P., Landín M. (2019). Current Stage of Marine Ceramic Grafts for 3D Bone Tissue Regeneration. Mar. Drugs.

[11] Gao Y., Jin X. (2019). Dual Crosslinked Methacrylated Alginate Hydrogel Micron Fibers and Tissue Constructs for Cell Biology. Mar. Drugs.

[12] Shaala L.A., Asfour H.Z., Youssef D.T., Żółtowska-Aksamitowska S., Wysokowski M., Tsurkan M., Galli R., Meissner H., Petrenko I., Tabachnick K. (2019). New source of 3D chitin scaffolds: The Red Sea demosponge Pseudoceratina arabica (Pseudoceratinidae, Verongiida). Mar. Drugs.

[13] Bardakova K.N., Akopova T.A., Kurkov A.V., Goncharuk G.P., Butnaru D.V., Burdukovskii V.F., Antoshin A.A., Farion I.A., Zharikova T.M., Shekhter A.B. (2019). From Aggregates to Porous Three-Dimensional Scaffolds through a Mechanochemical Approach to Design Photosensitive Chitosan Derivatives. Mar. Drugs.

[14] Adnen N.A.I., Halim N.A.A., Nor M.A.A.M. (2017). Development of Hydroxyapatite from Setiu Coral via Hydrothermal Method. AIP Conf. Proc..

[15] Shavandi A., Bekhit A.E.-D.A., Ali A., Sun Z. (2015). Synthesis of nano-hydroxyapatite (nHA) from waste mussel shells using a rapid microwave method. Mater. Chem. Phys..

[16] Rujitanapanich S., Kumpapan P., Wanjanoi P. (2014). Synthesis of hydroxyapatite from oyster shell via precipitation. Energy Procedia.

[17] Raya I., Mayasari E., Yahya A., Syahrul M., Latunra A.I. (2015). Shynthesis and characterizations of calcium hydroxyapatite derived from crabs shells (Portunus pelagicus) and its potency in safeguard against to dental demineralizations. Inter. J. Biomater..

[18] Mohandes F., Salavati-Niasari M. (2014). Simple morphology-controlled fabrication of hydroxyapatite nanostructures with the aid of new organic modifiers. Chem. Eng. J..

[19] Mohandes F., Salavati-Niasari M. (2014). Particle size and shape modification of hydroxyapatite nanostructures synthesized via a complexing agent-assisted route. Mater. Sci. Eng. C Mater. Biol. Appl..

[20] Wu S.-C., Hsu H.-C., Wu Y.-N., Ho W.-F. (2011). Hydroxyapatite synthesized from oyster shell powders by ball milling and heat treatment. Mater. Charact..

[21] Singh A. (2012). Hydroxyapatite, a biomaterial: Its chemical synthesis, characterization and study of biocompatibility prepared from shell of garden snail, Helix aspersa. Bull. Mater. Sci..

[22] Santhosh S., Balasivanandha Prabu S. (2012). Characterization of Hydroxyapatite Synthesized from Sea Shells and Electrospin Coating of Hydroxyapatite for Biomedical Applications. Advanced Materials Research.

[23] Kuboki Y., Takita H., Kobayashi D., Tsuruga E., Inoue M., Murata M., Nagai N., Dohi Y., Ohgushi H. (1998). BMP-induced osteogenesis on the surface of hydroxyapatite with geometrically feasible and nonfeasible structures: Topology of osteogenesis. J. Biomed. Mater. Res..

[24] Maji K., Dasgupta S., Pramanik K., Bissoyi A. (2016). Preparation and evaluation of gelatin-chitosan-nanobioglass 3D porous scaffold for bone tissue engineering. Inter. J. biomater..

[25] Zahedi E., Esmaeili A., Eslahi N., Shokrgozar M., Simchi A. (2019). Fabrication and Characterization of Core-Shell Electrospun Fibrous Mats Containing Medicinal Herbs for Wound Healing and Skin Tissue Engineering. Mar. Drugs.

[26] Szatkowski T., Kołodziejczak-Radzimska A., Zdarta J., Szwarc-Rzepka K., Paukszta D., Wysokowski M., Ehrlich H., Jesionowski T. (2015). Synthesis and characterization of hydroxyapatite/chitosan composites. Physicochem. Probl. Miner. Process..

[27] Ehrlich H., Hanke T., Simon P., Born R., Fischer C., Frolov A., Langrock T., Hoffmann R., Schwarzenbolz U., Henle T. (2010). Carboxymethylation of collagen with respect to Ca-phosphate phases formation. J. Biomed. Mater. Res. B Appl. Mater..

[28] Ehrlich H., Hanke T., Born R., Fischer C., Frolov A., Langrock T., Hoffmann R., Schwarzenbolz U., Henle T., Simon P. (2009). Mineralization of biomimetically carboxymethylated collagen fibrils in a model dual membrane diffusion system. J. Membr. Sci..

[29] Ehrlich H., Hanke T., Frolov A., Langrock T., Hoffmann R., Fischer C., Schwarzenbolz U., Henle T., Born R., Worch H. (2009). Modification of collagen in vitro with respect to formation of Nɛ-carboxymethyllysine. Int. J. Biol. Macromol..

[30] Pompe W., Worch H., Habraken W.J., Simon P., Kniep R., Ehrlich H., Paufler P. (2015). Octacalcium phosphate–a metastable mineral phase controls the evolution of scaffold forming proteins. J. Mater. Chem. B.

[31] Stancu I.C., Vasile D.M.D.E., Trusca R., Antoniac I., Vasilescu D.S. (2011). Porous calcium alginate-gelatin interpenetrated matrix and its biomineralization potential. J. Mater. Sci. Mater. Med..

[32] Eslahi N., Mahmoodi A., Mahmoudi N., Zandi N., Simchi A. (2019). Processing and Properties of Nanofibrous Bacterial Cellulose-Containing Polymer Composites: A Review of Recent Advances for Biomedical Applications. Polym. Rev..

[33] Devi N., Maji T.K. (2009). Preparation and evaluation of gelatin/sodium carboxymethyl cellulose polyelectrolyte complex microparticles for controlled delivery of isoniazid. AAPS PharmSciTech.

[34] Sartuqui J., Gravina A.N., Rial R., Benedini L.A., Yahia L.H., Ruso J.M., Messina P.V. (2016). Biomimetic fiber mesh scaffolds based on gelatin and hydroxyapatite nano-rods: Designing intrinsic skills to attain bone reparation abilities. Colloids Surf. B Biointerfaces.

[35] Ernsting M.J., Murakami M., Undzys E., Aman A., Press B., Li S.-D. (2012). A docetaxel-carboxymethylcellulose nanoparticle outperforms the approved taxane nanoformulation, Abraxane, in mouse tumor models with significant control of metastases. J. Control. Release.

[36] Ernsting M.J., Foltz W.D., Undzys E., Tagami T., Li S.-D. (2012). Tumor-targeted drug delivery using MR-contrasted docetaxel–carboxymethylcellulose nanoparticles. Biomaterials.

[37] Capanema N.S., Mansur A.A., de Jesus A.C., Carvalho S.M., de Oliveira L.C., Mansur H.S. (2018). Superabsorbent crosslinked carboxymethyl cellulose-PEG hydrogels for potential wound dressing applications. Int. J. Biol. Macromol..

[38] Wake M.C., Patrick C.W., Mikos A.G. (1994). Pore morphology effects on the fibrovascular tissue growth in porous polymer substrates. Cell Transplant..

[39] Annabi N., Nichol J.W., Zhong X., Ji C., Koshy S., Khademhosseini A., Dehghani F. (2010). Controlling the porosity and microarchitecture of hydrogels for tissue engineering. Tissue Eng. Part B Rev..

[40] Uehara M., Li X., Sheikhi A., Zandi N., Walker B., Saleh B., Banouni N., Jiang L., Ordikhani F., Dai L. (2019). Anti-IL-6 eluting immunomodulatory biomaterials prolong skin allograft survival. Sci. Rep..

[41] Ghaffari R., Eslahi N., Tamjid E., Simchi A. (2018). Dual-sensitive hydrogel nanoparticles based on conjugated thermoresponsive copolymers and protein filaments for triggerable drug delivery. ACS Appl. Mater. Interfaces.

[42] Kurosawa Y., Sato S., Okuyama T., Taoka M. (2019). Sequential two-step chromatographic purification of infectious poliovirus using ceramic fluoroapatite and ceramic hydroxyapatite columns. PLoS ONE.

[43] Tan Q., Li S., Ren J., Chen C. (2011). Fabrication of porous scaffolds with a controllable microstructure and mechanical properties by porogen fusion technique. Int. J. Mol. Sci..

[44] Kaplan D.L. (1998). Mollusc shell structures: Novel design strategies for synthetic materials. Curr. Opin. Solid State Mater. Sci..

[45] Mohandes F., Salavati-Niasari M., Fereshteh Z., Fathi M. (2014). Novel preparation of hydroxyapatite nanoparticles and nanorods with the aid of complexing agents. Ceram. Int..

[46] Smičiklas I., Onjia A., Raičević S. (2005). Experimental design approach in the synthesis of hydroxyapatite by neutralization method. Sep. Purif. Technol..

[47] Dubey P., Sharma V., Mitra S., Verma G., Hassan P., Dutta B., Johnson M., Mukhopadhyay R. (2017). Nanoscopic dynamics in hybrid hydroxyapatite-CTAB composite. J. Appl. Phys..

[48] Sankar S., Kumar Ramajayam K., Thirugnanam A. (2016). A novel method to fabricate porous tricalcium phosphate composite scaffolds for bone tissue engineering applications. Mater. Technol..

[49] Baniasadi H., Mashayekhan S., Fadaoddini S., Haghirsharifzamini Y. (2016). Design, fabrication and characterization of oxidized alginate–gelatin hydrogels for muscle tissue engineering applications. J. Biomater. Appl..

[50] Buhus G., Popa M., Desbrieres J. (2009). Hydrogels based on carboxymethylcellulose and gelatin for inclusion and release of chloramphenicol. J. Bioact. Compat. Polym..

[51] Divieto C., Sassi M.P. (2015). A first approach to evaluate the cell dose in highly porous scaffolds by using a nondestructive metabolic method. Future Sci. OA.

[52] Guerrini L., Alvarez-Puebla R.A., Pazos-Perez N. (2018). Surface modifications of nanoparticles for stability in biological fluids. Materials.

[53] Moroi Y., Motomura K., Matuura R. (1974). The critical micelle concentration of sodium dodecyl sulfate-bivalent metal dodecyl sulfate mixtures in aqueous solutions. J. Colloid Interface Sci..

[54] Ren F., Leng Y., Xin R., Ge X. (2010). Synthesis, characterization and ab initio simulation of magnesium-substituted hydroxyapatite. Acta Biomater..

[55] Li W., Zhang M., Zhang J., Han Y. (2006). Self-assembly of cetyl trimethylammonium bromide in ethanol-water mixtures. Front. Chem. China.

[56] Wu S.-H., Chen D.-H. (2004). Synthesis of high-concentration Cu nanoparticles in aqueous CTAB solutions. J. Colloid Interface Sci..

[57] Mohandes F., Salavati-Niasari M. (2014). In vitro comparative study of pure hydroxyapatite nanorods and novel polyethylene glycol/graphene oxide/hydroxyapatite nanocomposite. J. Nanopart. Res..

[58] Thadavirul N., Pavasant P., Supaphol P. (2014). Development of polycaprolactone porous scaffolds by combining solvent casting, particulate leaching, and polymer leaching techniques for bone tissue engineering. J. Biomed. Mater. Res. Part. A.

[59] Haiying Y., Howard W., Paul H., Shang-you Y. (2008). Effect of Porosity and Pore Size on Microstructures and Mechanical Properties of Poly-e-Caprolactone-Hydroxyapatite Composites. J. Biomed. Mater. Res. B Appl. Biomater..

[60] Murphy W.L., Dennis R.G., Kileny J.L., Mooney D.J. (2002). Salt fusion: An approach to improve pore interconnectivity within tissue engineering scaffolds. Tissue Eng..

[61] Ren J., Zhao P., Ren T., Gu S., Pan K. (2008). Poly (D, L-lactide)/nano-hydroxyapatite composite scaffolds for bone tissue engineering and biocompatibility evaluation. J. Mater. Sci. Mater. Med..

[62] Karageorgiou V., Kaplan D. (2005). Porosity of 3D biomaterial scaffolds and osteogenesis. Biomaterials.

[63] Mohandes F., Bakhtiar H., Nekoofar M., Ostad S.N., Simchi A. (2019). Preparing Hydroxyapatite Nanostructures. US Patent.

[64] Guarino V., Causa F., Taddei P., Di Foggia M., Ciapetti G., Martini D., Fagnano C., Baldini N., Ambrosio L. (2008). Polylactic acid fibre-reinforced polycaprolactone scaffolds for bone tissue engineering. Biomaterials.

